# Utility of an image fusion system for 3D preoperative planning and fluoroscopy in the osteosynthesis of distal radius fractures

**DOI:** 10.1186/s13018-019-1370-z

**Published:** 2019-11-06

**Authors:** Yuichi Yoshii, Yasukazu Totoki, Satoshi Sashida, Shinsuke Sakai, Tomoo Ishii

**Affiliations:** 1 Department of Orthopaedic Surgery, Tokyo Medical University Ibaraki Medical Center, 3-20-1 Chuo, Ami, Inashiki, Ibaraki 300-0395 Japan; 2 Department of Orthopaedic Surgery, University of Tsukuba Hospital, Tsukuba, Ibaraki 305-8576 Japan; 3 LEXI Co. Ltd., Sugamo, Tokyo, 170-0002 Japan

**Keywords:** Image fusion, Preoperative plan, Fluoroscopy, Distal radius fracture, Computed tomography, Osteosynthesis

## Abstract

**Background:**

Recently, computerized virtual surgery planning has been increasingly applied in various orthopedic procedures. In this study, we developed an image fusion system for 3D preoperative planning and fluoroscopy for the osteosynthesis. To assess the utility of image fusion system, we evaluated the reproducibility of preoperative planning in the osteosynthesis of distal radius fractures with using the image fusion system, and compared with the reproducibility of the patients without using the image fusion system.

**Methods:**

Forty-two wrists of 42 distal radius fracture patients who underwent osteosynthesis using volar locking plates were evaluated. The patients were divided into two groups. Image fusion group utilized three-dimensional (3D) preoperative planning and image fusion system. Control group utilized only 3D preoperative planning. In both groups, 3D preoperative planning was performed in order to determine reduction, placement, and choice of implants. In the image fusion group, the outline of planned image was displayed on a monitor overlapping with fluoroscopy images during surgery. Reductions were evaluated by volar tilt and radial inclination of 3D images. Plate positions were evaluated with distance to joint surface, plate center axis position, and inclination relative to the radius axis. Screw choices were recorded for the plan and actual choices for each screw hole. Differences in the parameters between pre- and postoperative images were evaluated. Differences in reduction shape, plate positions, and screw choices were compared between groups.

**Results:**

The differences in the distance from plate to joint surface were significantly smaller in the image fusion group compared to the control group (*P* < 0.01). The differences in the distal screw choices were significantly smaller in the image fusion group compared to the control group (*P* < 0.01).

**Conclusions:**

The image fusion system was useful to reproduce the planned plate position and distal screw choices in the osteosynthesis of distal radius fractures.

**Trial registration:**

ClinicalTrials.gov, NCT03764501

## Background

The main points for the treatment of fractures are to acquire anatomical reduction and to reconstruct a biomechanically stable joint in relation to the diaphysis with correct alignment of the axis and rotation. To achieve anatomical reduction and appropriate implant choices in the osteosynthesis of fractures, preoperative planning is useful. Generally, preoperative planning has been performed by handwriting hardcopy radiographs along with tracing paper or simple measurements of picture archiving and communication systems. However, sometimes, it was difficult to determine the rotational reduction and appropriate implant placements/choices preoperatively. In addition, there are inaccuracies due to differences in the scale of radiographic images.

Recently, computerized virtual surgery planning has been increasingly applied in various orthopedic procedures. In fact, three-dimensional (3D) preoperative planning and intraoperative navigation systems are clinically utilized for fracture management [1–5]. While it is unclear whether or not the use of these methods improves clinical outcomes, there may be some benefits for fracture reduction and less extensive dissection because of a better understanding of fracture shape [6]. 3D preoperative planning is good for preprocessing visualization, permitting viewing and understanding of the fracture displacement, and for manipulation images. In a previous study, we developed a 3D preoperative software for fracture management [7]. It was found that the software was useful for visualizing and planning treatment of fractures and choice of implants. However, there was no way to compare the planning image to the fluoroscopic image directly during surgery. Therefore, we made another step forward and promoted development of an image fusion system for the 3D preoperative planning and fluoroscopy. In this study, we hypothesized that the use of an image fusion system would improve the reproducibility of the reduction shape and implant placements/choices. Distal radius fracture is one of the most frequent fractures in the human body [8–12]. In the treatment of distal radius fracture, the surgical indication has tended to increase because of improvements in internal fixation materials [13–16]. In this study, we assessed the utility of an image fusion system by evaluating the reproducibility of preoperative planning in the osteosynthesis of distal radius fractures. In addition, the reproducibility was compared with the postoperative results of patients treated without using an image fusion system.

## Methods

This study protocol was approved by the Institutional Review Board (no. 14-21). This was a randomized controlled trial using block randomization (levels of evidence II). This study was registered as NCT03764501 at ClinicalTrials.gov. This study was performed in accordance with the relevant guidelines and regulations. Informed consent was obtained from all individual participants included in the study. Forty-two wrists of 42 distal radius fracture patients who underwent osteosynthesis using volar locking plates (32 females, 10 males, mean age 63.3 years, age range 19–91) were evaluated. Patients were excluded if they reported a previous history of traumatic arm injuries. The patients were divided into two groups. The image fusion group (*n* = 21) utilized 3D preoperative planning and an image fusion system for osteosynthesis. The control group (*n* = 21) utilized only 3D preoperative planning. According to the preoperative CT scans, fractures were classified using the AO classification system. Patients with a common age group, sex, and fracture type were assigned to each group.

### Preoperative planning

In both groups, 3D digital preoperative planning and a surgical simulation were performed prior to the surgery. The details of the simulation steps were described previously [7]. Reduction and placement of the implants were simulated using software developed by the authors (Zed-Trauma wrist version, LEXI Co., Ltd. Tokyo, Japan). CT with contiguous images of 1 mm thickness was used for the simulation. After importing the DICOM images to the software, a 3D image of the distal radius was made. Each distal radius fracture was segmented according to the fracture fragments using the cut function. Each fragment was repositioned in accordance with fracture lines. After repositioning the fragments, the bone shape was checked 3D. Reduction of the fragment was performed to regain the volar tilt and radial inclination, with a less than 2 mm step-off for the intra-articular displacement referring to a healthy side wrist X-ray. If there was fracture comminution, the fragments were separated based on up to 5 mm bone fragments. In the second step, simulations of the volar locking plate implantation with various sizes of plates and screws were performed. Stellar II locking plates (HOYA Technosurgical, Inc., Tokyo, Japan) were used in this study. This plate system has small, medium, and large sizes for the width, and short and long sizes for the plate length. Screw lengths from 10 to 24 mm for the distal (2.4 mm diameter) and 10 to 20 mm for the proximal (2.6 mm diameter) are available. Computer-aided design models of different-sized implants were installed in the software. The plate size was chosen to cover the distal fragment maximally and not exceed the width of the distal radius. The screw lengths for each screw hole were determined. All patients had pre- and postoperative CT scans in order to compare the planned and postoperative reduction shape and placement of the implant.

### Image fusion system

**Fig. 1:**
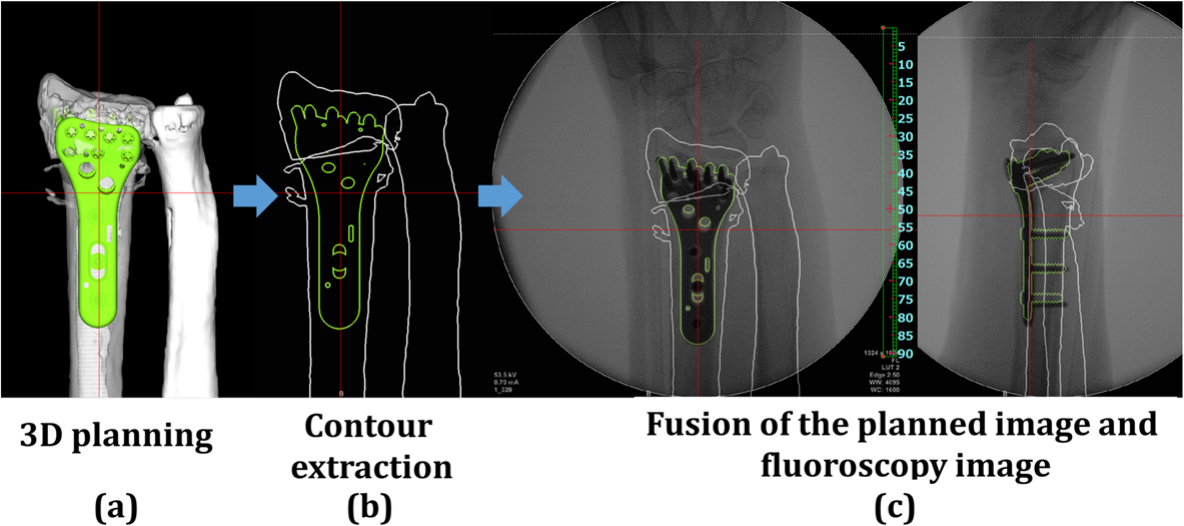
Image fusion system. **a** 3D preoperative planning image. **b** Contour extraction of 3D image. **c** Examples of fusion images. Fusion images were created by overlapping the outline of the 3D preoperative plan and the fluoroscopic image

An image fusion system was developed through this study and installed in the computer. This system allows us to visualize the overlapping images of the preoperative plan and fluoroscopy during surgery (Fig. 1). The 3D images of the preoperative plans were converted to the digitally reconstructed radiographs (DRR). Based on 3D images, bones and implants contour extraction images are displayed. Fusion images were displayed on the monitor with overlapping the outline of the 3D preoperative plan and the fluoroscopic image. In this study, we created the outlines of anterior-posterior view, lateral view, and axial view. According to the direction of the fluoroscopic image, the directions of the outline image were changed to an anterior-posterior view, a lateral view, and an axial view.

### Surgical intervention

After the preoperative planning, osteosynthesis was performed under general anesthesia. In the image fusion group, the outline of the planned image was displayed on a monitor overlapping the fluoroscopy image during surgery. Before the surgery, the image size was calibrated by a measurement of known length. Surgeons performed osteosynthesis with the fusion image. The surgeons tried to reproduce the planned shape of the reduction and the position of the implant by checking the distances from the margin of the implant to the margin of the radius under fluoroscopy. In the control group, the operator performed the reduction and the placement of the plate while comparing images between the preoperative plan and fluoroscopy during surgery. In both groups, the screw sizes were determined by intraoperative measurement referring to the preoperative plan. The surgeries were performed by several orthopedic surgeons along with a hand surgeon.

### Evaluations

To evaluate the accuracy of the reduction, preoperative planning and postoperative reductions were compared by measuring the volar tilt and radial inclination of the 3D images in both groups. The axis of the radius was adjusted. The angle between a line from the dorsal edge to the volar edge of the radius and a line perpendicular to the longitudinal axis of the radius was measured as 3D volar tilt in the sagittal view. The angle between a line from the radial styloid tip to the ulnar aspect of the distal radius and a line perpendicular to the longitudinal axis of the radius was measured as the 3D radial inclination in the coronal view.

**Fig. 2:**
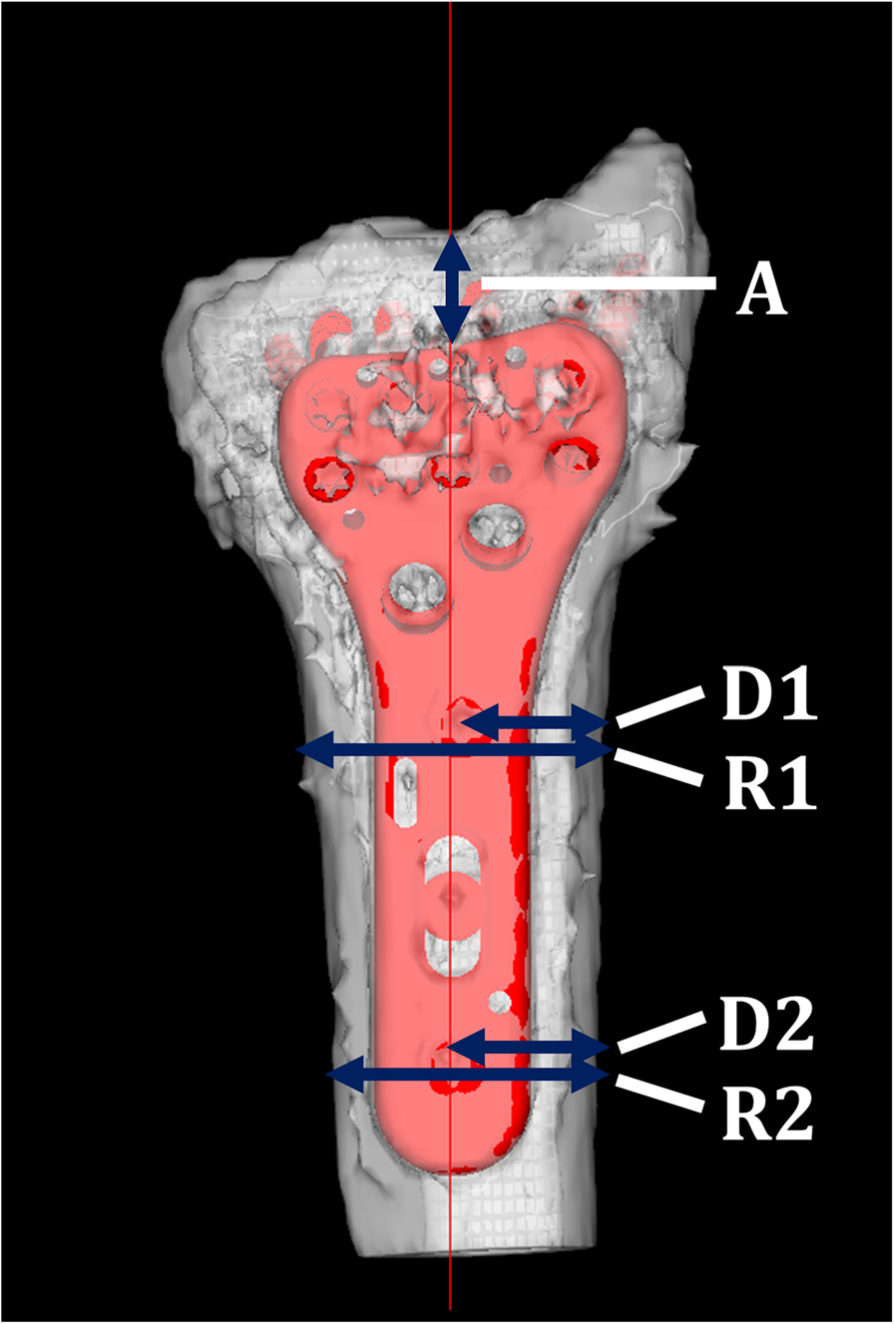
Evaluation of plate positions. A: distance from the plate to the joint surface. D1, D2: distance from the plate center to the radial edge of the distal radius. R1, R2: transverse diameter of the radius

Plate positions were evaluated by distance to the joint surface, plate center axis position, and inclination relative to the radius axis (Fig. 2). The distance from the plate to the joint surface was defined by the distance from the distal edge of the plate and the distal radius joint surface at the longitudinal axis of the plate (= A). For the plate center axis position and the inclination relative to the radius axis, the distance from the plate center to the radial edge of the distal radius and the transverse diameter of the radius were measured at the first and third proximal screw hole levels. The mean of the center position relative to the radius at the first and the third screw hole levels was defined as the plate center axis position (= (D1/R1 + D2/R2)/2). The difference in the plate center position between the first and third proximal screw hole levels was defined as the inclination (= D1/R1 − D2/R2). Screw choices were recorded for the plan and actual choices for each screw hole. The screw holes were numbered from 1 to 8 for the distal (there were 7 holes in the small and medium plates, and 8 holes in the large plate), and from 1 to 4 for the proximal (there were 3 holes in the short plate, and 4 holes in the long plate). The screw lengths actually chosen were recorded according to the screw number.

### Statistical analysis

To measure the reduction accuracy, the differences in the reduction shape (volar tilt and radial inclination) between the preoperative plans and the postoperative reductions were evaluated. To assess the accuracy of the implant placement, the differences in the plate placement (distance, plate center axis position, and inclination relative to the radius axis) between the preoperative plans and the postoperative reductions were evaluated. As for the accuracy of the screw choices, the differences in the screw lengths between the preoperative plans and the actual choices for each screw hole were evaluated. The results are expressed as the mean ± standard deviation. The absolute values of the parameters were compared between the image fusion group and the control group. Welch’s *t* test was used for a comparison of the differences. *P* values of < 0.05 were considered significant. All analyses were performed using BellCurve for Excel version 2.12 (SSRI Co., Tokyo, Japan).

## Results

**Fig. 3:**
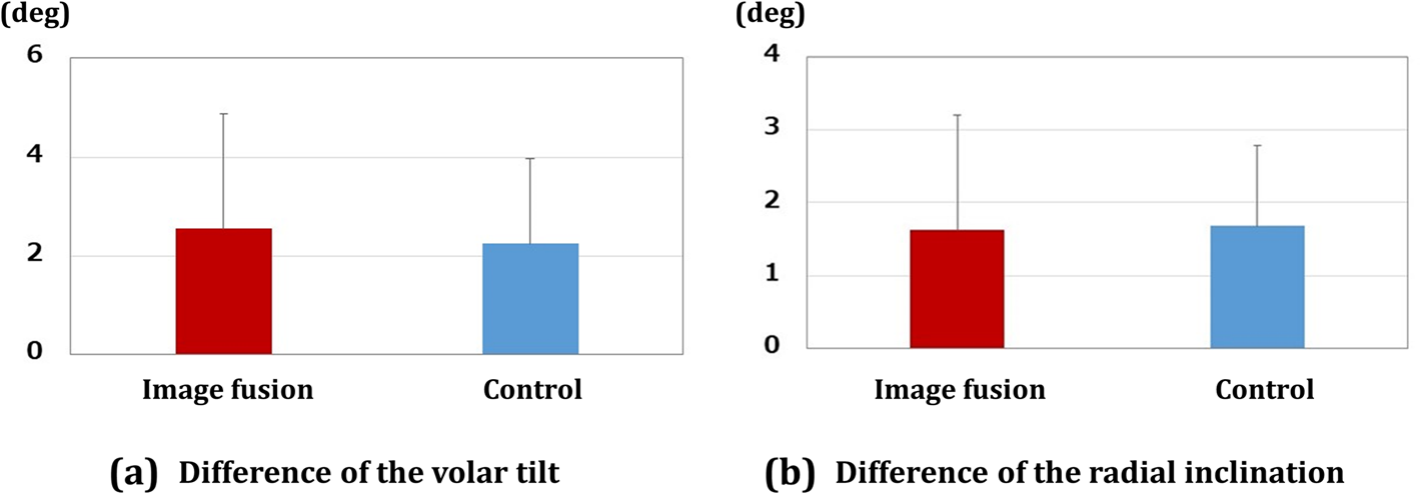
Results for reduction accuracy. **a** Difference in the volar tilt. **b** Difference in the radial inclination. Red bar shows the results for the image fusion group, and blue bar those for the control group

There were six patients with A3 type fracture, nine patients with C2 type fracture, and six patients with C3 type fracture in each group. The results of reduction accuracy are shown in Fig. 3. The differences in the volar tilt between the preoperative plan and the postoperative results were 2.5 ± 2.3° and 2.2 ± 1.7° for the image fusion group and control group, respectively. The differences in the radial inclination between the preoperative plans and the postoperative results were 1.6 ± 1.6° and 1.7 ± 1.1° for the image fusion group and control group, respectively. There were no significant differences in the reduction accuracy between the groups.

**Fig. 4:**
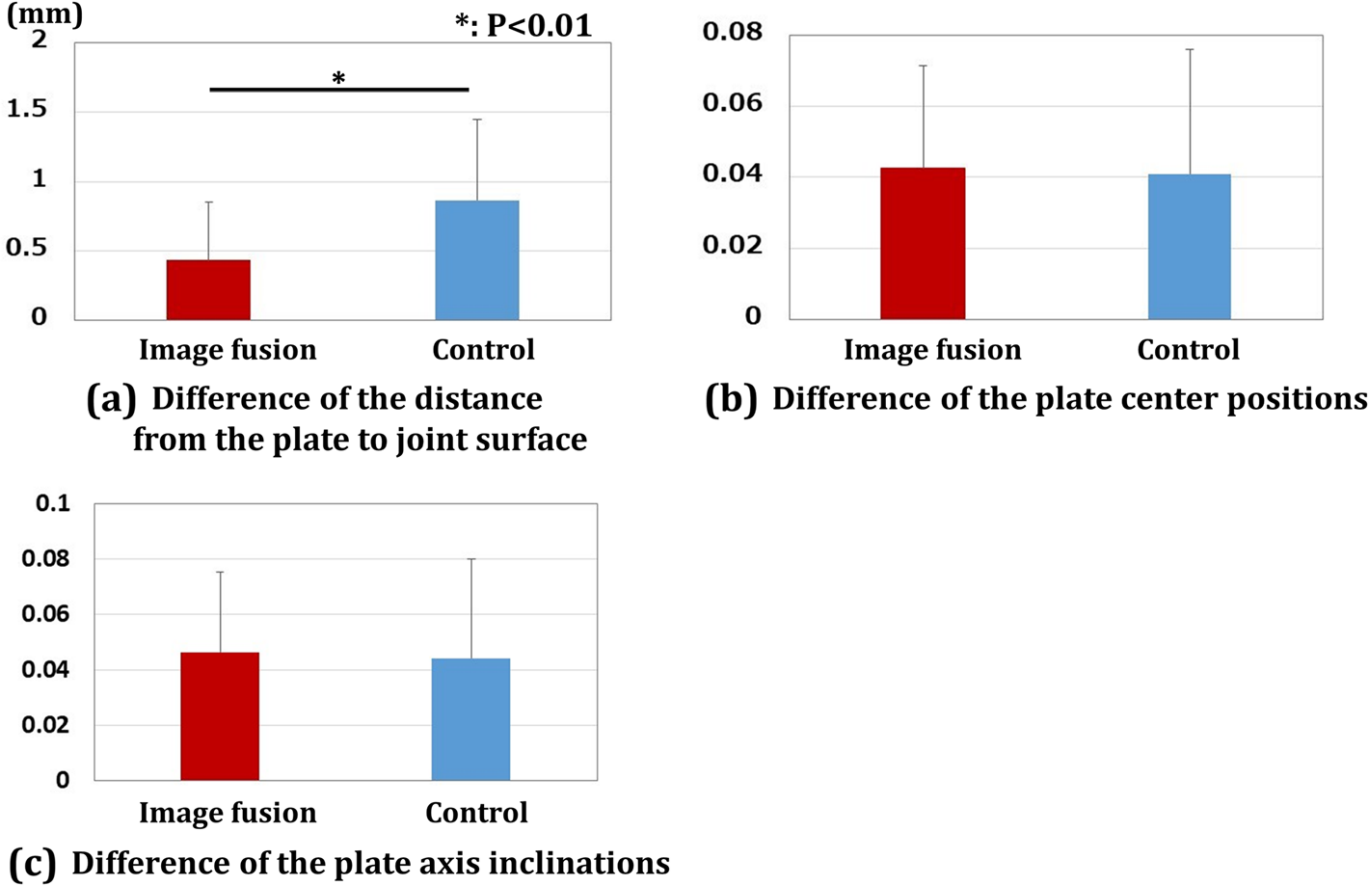
Results for plate positions. **a** Difference in the distance from the plate to the joint surface. There was a significant difference between the image fusion group and the control group (*P < 0.01). **b** Difference in plate center positions. **c** Difference in plate axis inclinations. Red bar shows the results for the image fusion group, and the blue bar shows those for the control group

The results of plate positions are shown in Fig. 4. The planned plate sizes were used for all patients in both groups. The differences in the distance from the plate to the joint surface were 0.43 ± 0.42 mm and 0.86 ± 0.59 mm for the image fusion group and control group, respectively. The differences in the plate center positions were 0.04 ± 0.02 and 0.04 ± 0.03 for the image fusion group and control group, respectively. The differences in the plate axis inclinations were 0.05 ± 0.03 and 0.04 ± 0.04 for the image fusion group and control group, respectively. For the distance from the plate to the joint surface, there were significantly smaller differences in the image fusion group compared to the control group (*P* < 0.01).

**Fig. 5:**
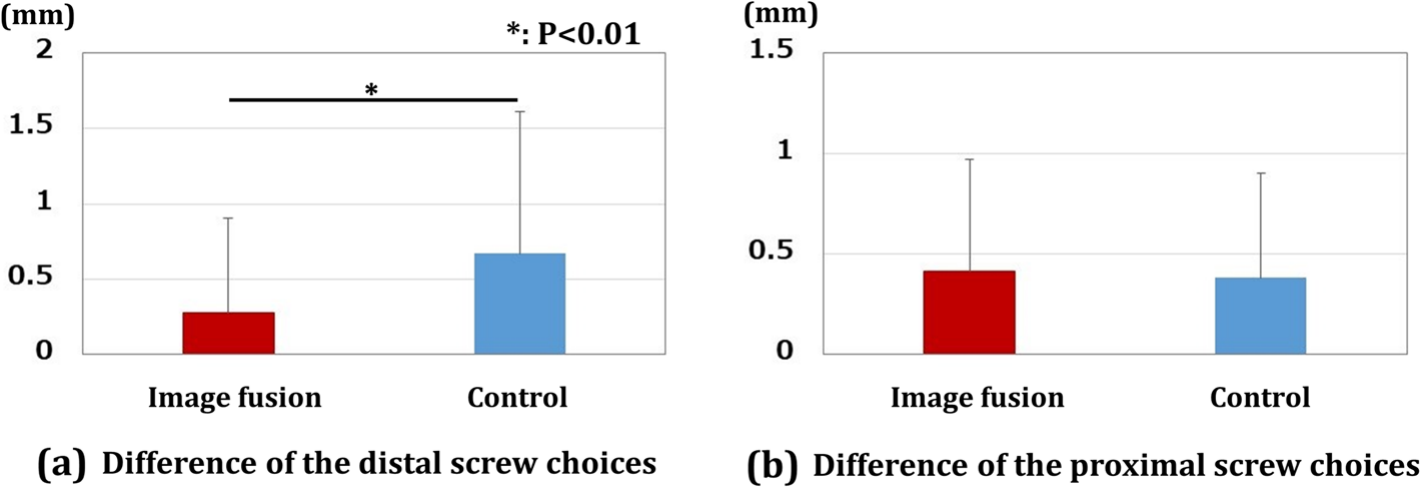
Results for screw choices. **a** Difference in the distal screw choices. There was a significant difference between the image fusion group and the control group (*P < 0.01). **b** Difference in the proximal screw choices. Red bar shows the results for the image fusion group, and the blue bar shows those for the control group

The results of screw choices are shown in Fig. 5. The differences in the distal screw choices were 0.28 ± 0.62 mm and 0.67 ± 0.94 mm for the image fusion group and control group, respectively. The differences in the proximal screw choices were 0.41 ± 0.55 mm and 0.38 ± 0.52 mm for the image fusion group and control group, respectively. The differences in the distal screw choices were significantly smaller in the image fusion group compared to the control group (*P* < 0.01).

## Discussion

In this study, we investigated the utility of one next-generation method for 3D preoperative planning with an image fusion system in the treatment of distal radius fractures. The advantage of this system is that it enables simple navigation without introducing surgical navigation system. Once installed the application in the computer, the surgical support with image fusion is possible. In recent years, clinical applications of computer-assisted surgery have been widely reported in orthopedic surgery [17–20]. Computer-assisted surgery for artificial joints and in spine surgery is known to be useful and is becoming established [17–19]. On the other hand, the introduction of computer-aided technology for treating fractures has lagged behind because of there are various fracture types, implant choices, and difficulty in terms of stable intraoperative image rendering. 3D bone morphology evaluation, preoperative planning, and intraoperative navigation based on computer-aided technology appear to be an attractive approach to increase the accuracy of surgery and reduce complications. Osteosynthesis for fracture is one of the most frequent operations in orthopedic surgery. In order to restore the lost motor function, it is important to set an ideal reposition and appropriate fixation. Inappropriate repositioning and internal fixation cause complications such as bone union failure, re-displacement of reduction position, and delayed recovery of the patient’s motor function. In order to prevent these complications, it is necessary to choose and place the optimal implants according to the individual fracture type and bone shape.

In a previous study, we attempted to introduce computer-aided technologies to treat fractures by developing and clinically applying 3D preoperative planning software based on CT images of fractures. It was found that a higher reduction accuracy and a reduction in the postoperative correction loss could be obtained by making a 3D preoperative plan [21]. However, there were no means to connect the 3D preoperative planning image directly to the fluoroscopic image during the surgery. Therefore, we developed an image fusion system for 3D preoperative planning. Using the image fusion system, we could directly compare the planning image with fluoroscopy. It was found that the image fusion system provided better reproducibility of the plate placement for the distance to the joint surface, as well as for screw lengths. As positioning of the volar locking plate is closely related to the biomechanical stability of the wrist joint [22, 23], good reproducibility of the planned positions of the plates is important. The plate needs to be positioned distally enough to provide sufficient fixation for the subchondral bone [24]. At the same time, it needs to avoid the distal screws penetrating into the joint. In addition, the length of distal locking screws is important because it is related to the fixation strength and complications [25, 26]. From these viewpoints, the image fusion system can be an effective tool to support better reproducibility for implant placement and screw choices.

There are some limitations to this study. First, there were no significant differences in the reduction accuracy, the plate axis position, or the inclination between the image fusion group and the control group. The reduction accuracies were almost the same levels for both groups. This suggests that even without using an image fusion system, the reductions were obtained at the same levels. In addition, the plate axis position and the inclination did not show any significant differences. These parameters were used for the first time to evaluate the reproducibility of plate axis positions. We still do not know if these parameters have clinical significance. As the appropriate coverage of the fragments was emphasized, improving the reproducibility of the parameters should be considered. Second, evaluations of impact on the clinical outcomes are still insufficient. The differences in the implant placement and choices may not affect the clinical outcomes. Third, the preoperative plan and the image fusion system require a CT scan. It is essential that the clinical justification for a CT scan be considered on an individual basis and that due consideration is given to the radiation risk and possible therapeutic benefit [27]. In addition, it is necessary to consider a method to reduce the radiation exposure dose. These points need to be elucidated in future studies.

In conclusion, we developed an image fusion system for 3D preoperative planning and fluoroscopy. The utility of the image fusion system was evaluated by the reproducibility of the preoperative plan in the osteosynthesis of distal radius fractures. It was found that the image fusion system provided better reproducibility of the plate placement for the distance to the joint surface and also for distal screw choices. This image fusion system may be useful to reproduce the planned plate position and screw choices in the osteosynthesis of distal radius fractures.

## Data Availability

The datasets used and/or analyzed during the current study are available from the corresponding author on reasonable request.

## Abbreviations

3D: Three-dimensional
DRR: Digitally reconstructed radiographs
